# Tangential excision of rhinophyma with electrocautery and healing by secondary intention: A case report

**DOI:** 10.1016/j.jpra.2026.08.028

**Published:** 2026-08-25

**Authors:** Ahmed AlBahlal, Lama A. Alkhwildi, Yara K. Alwathnani, Bassam Alawirdhi

**Affiliations:** a Plastic Surgery Department, Prince Sultan Military Medical City, Riyadh, Saudi Arabia; b College of Medicine, Imam Mohammad Ibn Saud Islamic University (IMSIU), Riyadh, Saudi Arabia; c Plastic and Reconstractive Surgery Department, King Saud Medical City, Riyadh, Saudi Arabia

**Keywords:** Rhinophyma, Tangential excision, Electrocautery, Sebaceous hyperplasia, Secondary intention, Rosacea

## Abstract

Rhinophyma is a benign, slowly progressive, disfiguring condition of the nose characterized by sebaceous gland hypertrophy, fibrosis, and follicular plugging, generally regarded as the end stage of phymatous rosacea. It predominantly affects middle-aged to elderly men and can cause significant cosmetic and psychosocial morbidity and, in advanced cases, nasal airway obstruction. We report a 71-year-old hypertensive man who presented with progressive, painless nasal enlargement involving the nasal tip and alar crests bilaterally. Incisional biopsy confirmed rhinophyma, showing sebaceous hyperplasia with cystic dilatation of the follicular infundibula and keratinous plugging. He underwent tangential excision using a cold blade combined with monopolar electrocautery, with hemostasis achieved by monopolar diathermy, and the wound was left to heal by secondary intention under a silver-based dressing. Healing was uneventful, with complete re-epithelialization and a satisfactory cosmetic outcome. This case supports tangential excision with monopolar electrocautery as a simple, effective, and reproducible technique for rhinophyma. Beyond the outcome itself, this report documents the intraoperative depth-control and symmetry cues used, the technical difficulties encountered during subunit contouring, and a staged postoperative wound-care protocol, so that an established but often summarily described technique can be reproduced in centres without access to laser or hydrosurgical platforms. A single case cannot establish superiority over alternative modalities, and comparative studies using standardized aesthetic outcome measures remain needed.

## Introduction

Rhinophyma is a chronic, progressive, disfiguring disorder of the nasal soft tissue characterized by sebaceous gland hypertrophy, fibrosis, telangiectasia, and follicular plugging, typically regarded as the late phymatous stage of rosacea.^1^ It may occur independently of overt rosacea, and not all rosacea progresses to phymatous change.^1^^,^^2^ It predominantly affects fair-skinned men in the fifth to seventh decades, with a male-to-female ratio of roughly 5:1 to 30:1, prompting speculation about a role of androgenic hormones.^3^ The pathogenesis is incompletely understood and likely multifactorial, involving chronic vasodilation, fluid extravasation, dermal edema, an augmented innate immune response, neurovascular dysregulation, sebaceous gland hyperplasia, and progressive fibrosis.^1^^,^^2^ Histologically it shows hypertrophic sebaceous glands, dilated follicular infundibula with keratinous plugging, dermal fibrosis, and a fibrovascular myxoid stroma. The lesion typically affects the lower two-thirds of the nose and is classified into glandular, fibrous, fibroangiomatous, and actinic variants, with severity scales such as Freeman’s used to guide treatment.^4^

Topical metronidazole, oral tetracyclines, or isotretinoin may help early, but established phymatous change is largely refractory to medical management and requires surgery.^1^^,^^5^ Described modalities include cold-steel tangential (decortication) excision, electrosurgery, dermabrasion, CO₂ and Er:YAG laser ablation, hydrosurgery, cryosurgery, and full-thickness excision with grafting or flap reconstruction.^3^, ^4^, ^5^, ^6^ Tangential excision with adjunctive electrocautery remains among the most popular, owing to its simplicity, low cost, preservation of pilosebaceous units permitting spontaneous re-epithelialization, and good aesthetic results.^7^ Nevertheless, the technique is more often named than described. Published accounts rarely specify how the depth of excision is controlled, which intraoperative landmarks are used to re-establish the aesthetic subunits, or how the open wound is managed during the weeks of secondary-intention healing, these are the steps that determine the result, and the steps a surgeon undertaking the procedure for the first time most needs to know. The gap is of practical consequence, because access to CO₂, hydrosurgery systems, and dermabrasion equipment remains restricted in many centres, so that the instrument-light option is often the only one available.^3^^,^^7^ We therefore report a case of moderate rhinophyma treated by tangential excision with monopolar electrocautery and secondary-intention healing, with the aim of documenting the operative sequence, the depth-control and symmetry cues applied intraoperatively, the technical difficulties encountered, and the structured postoperative wound-care protocol in sufficient detail to be reproduced.

## Case presentation

**Fig. 1:**
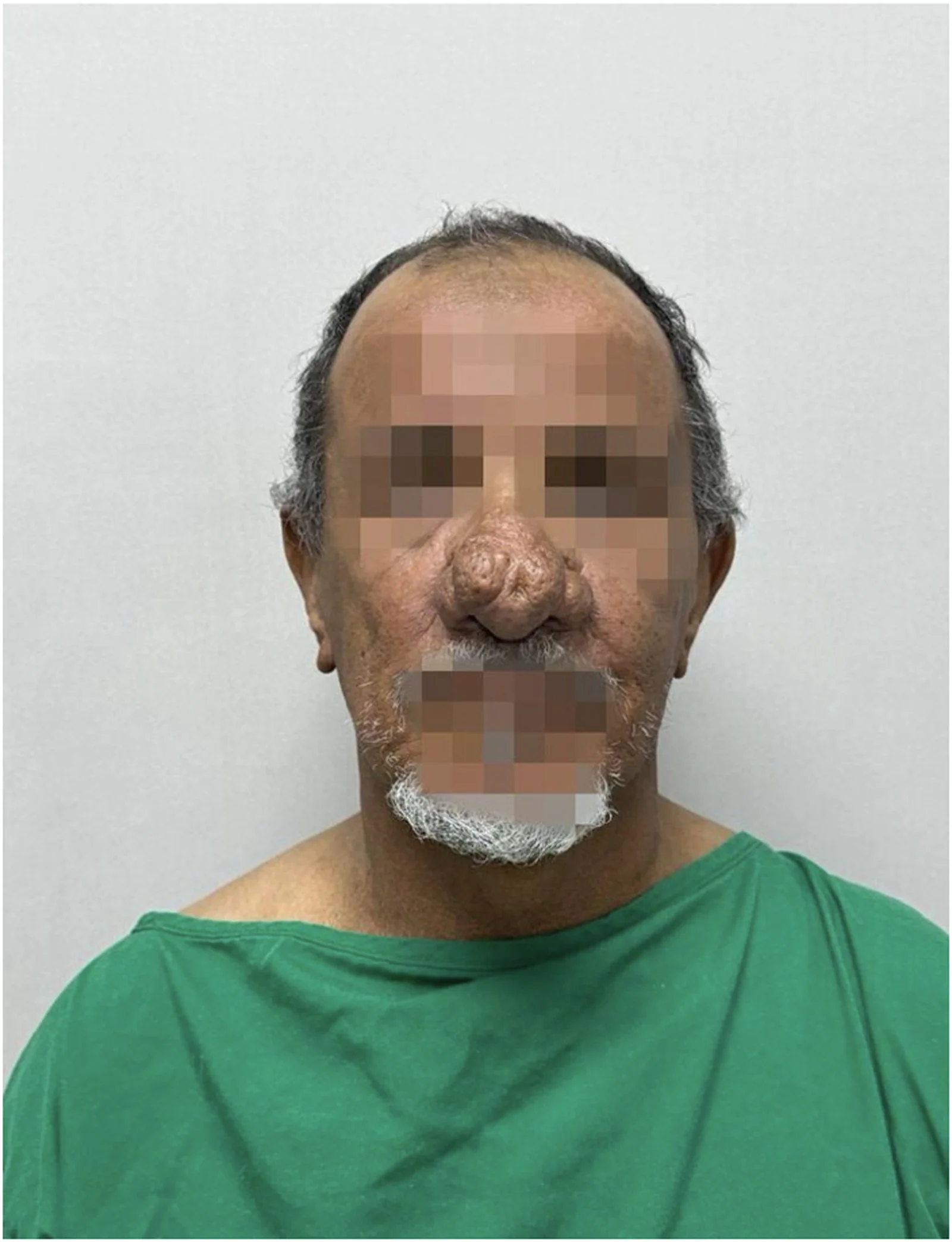
Preoperative frontal view showing moderate rhinophyma with lobulated, bulbous hypertrophy of the nasal tip and bilateral alar crests, with prominent pilosebaceous orifices and no active inflammation or ulceration.

A 71-year-old hypertensive man presented to the outpatient clinic with slowly progressive nasal enlargement evolving over several years. He denied pain, tenderness, bleeding, nasal obstruction, and systemic symptoms, and reported no facial flushing, recurrent erythema, or pustular eruptions and no prior treatment for the lesion. He denied alcohol abuse and was a non-smoker. Examination revealed soft, non-tender, lobulated hypertrophy of the nasal soft tissues centered over the nasal tip and extending laterally to the alar crest bilaterally (Fig. 1). The overlying skin showed prominent pilosebaceous orifices but no discoloration, ulceration, telangiectasia, or active inflammation. There was no nasal airway obstruction and no cervical lymphadenopathy, and the remainder of the head and neck examination was unremarkable. A working diagnosis of rhinophyma was made, and an incisional biopsy was performed before definitive surgery to exclude malignancy, particularly basal cell carcinoma.

The specimen, labeled rhinophyma, comprised multiple fragments of tan-to-brown skin measuring 5 × 4 × 0.5 cm in aggregate, with nodular cut surfaces. Microscopy showed marked sebaceous hyperplasia with cystic dilatation of the follicular infundibula and keratinous plugging, consistent with rhinophyma and without dysplasia or malignancy. The patient was then counseled regarding treatment options, the natural history of the disease, the tangential excision technique, expected secondary-intention healing, and the possibility of recurrence.

## Operative phase

In the operating theater under strict asepsis, the patient was supine with the head elevated. The nasal subunits were marked, with attention to the alar crests, alar grooves, and tip-defining points to preserve aesthetic landmarks. Local infiltration anesthesia using lidocaine with epinephrine was administered into the rhinophymatous tissue and along the infraorbital nerve distribution, providing analgesia and hemostatic vasoconstriction, and the face was prepped and draped sterilely.

**Fig. 2:**
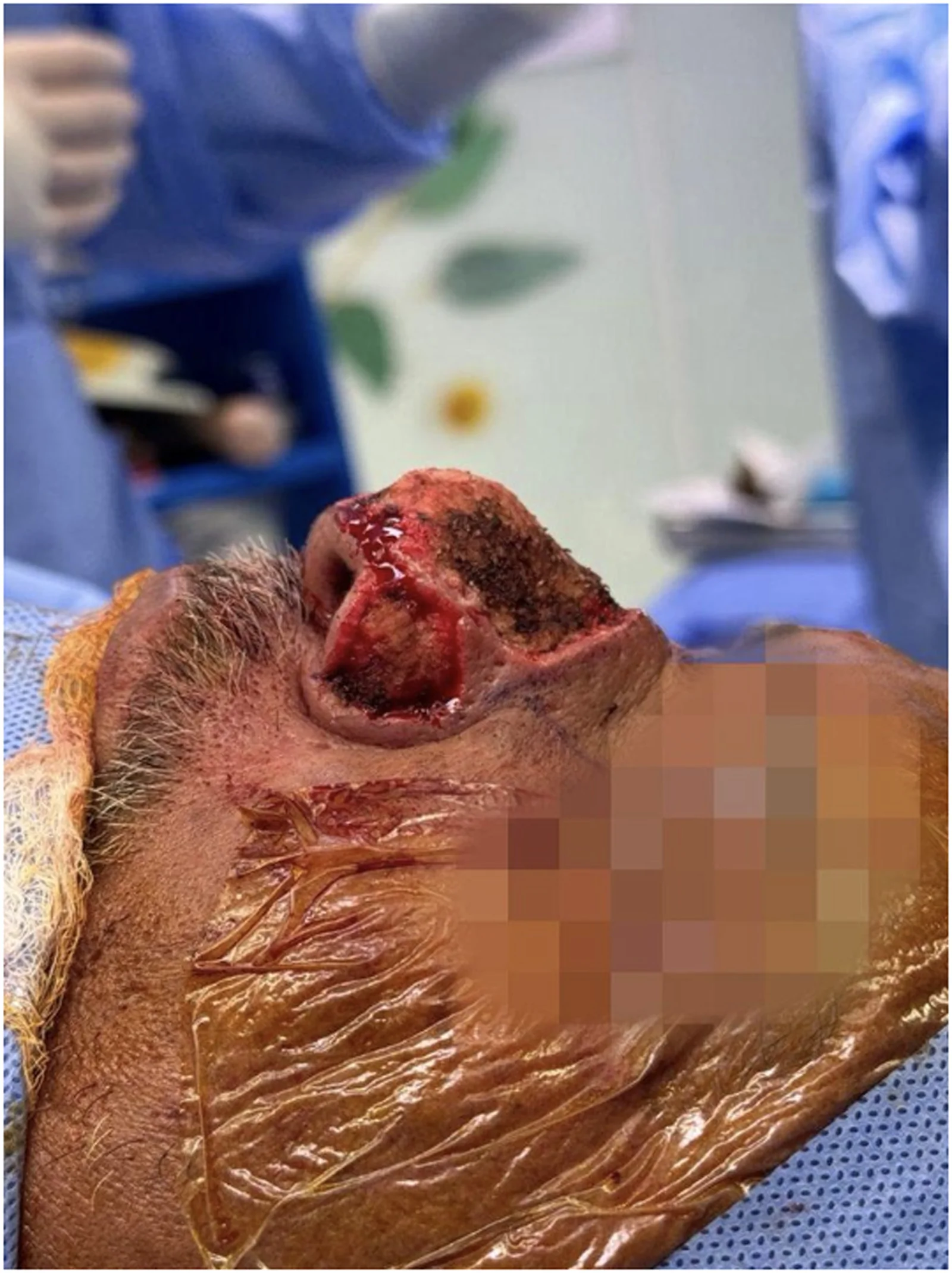
Intraoperative lateral view following tangential excision with a No 15 cold blade and monopolar electrocautery, demonstrating the debulked nasal tip with hemostasis achieved by focused diathermy.

**Fig. 3:**
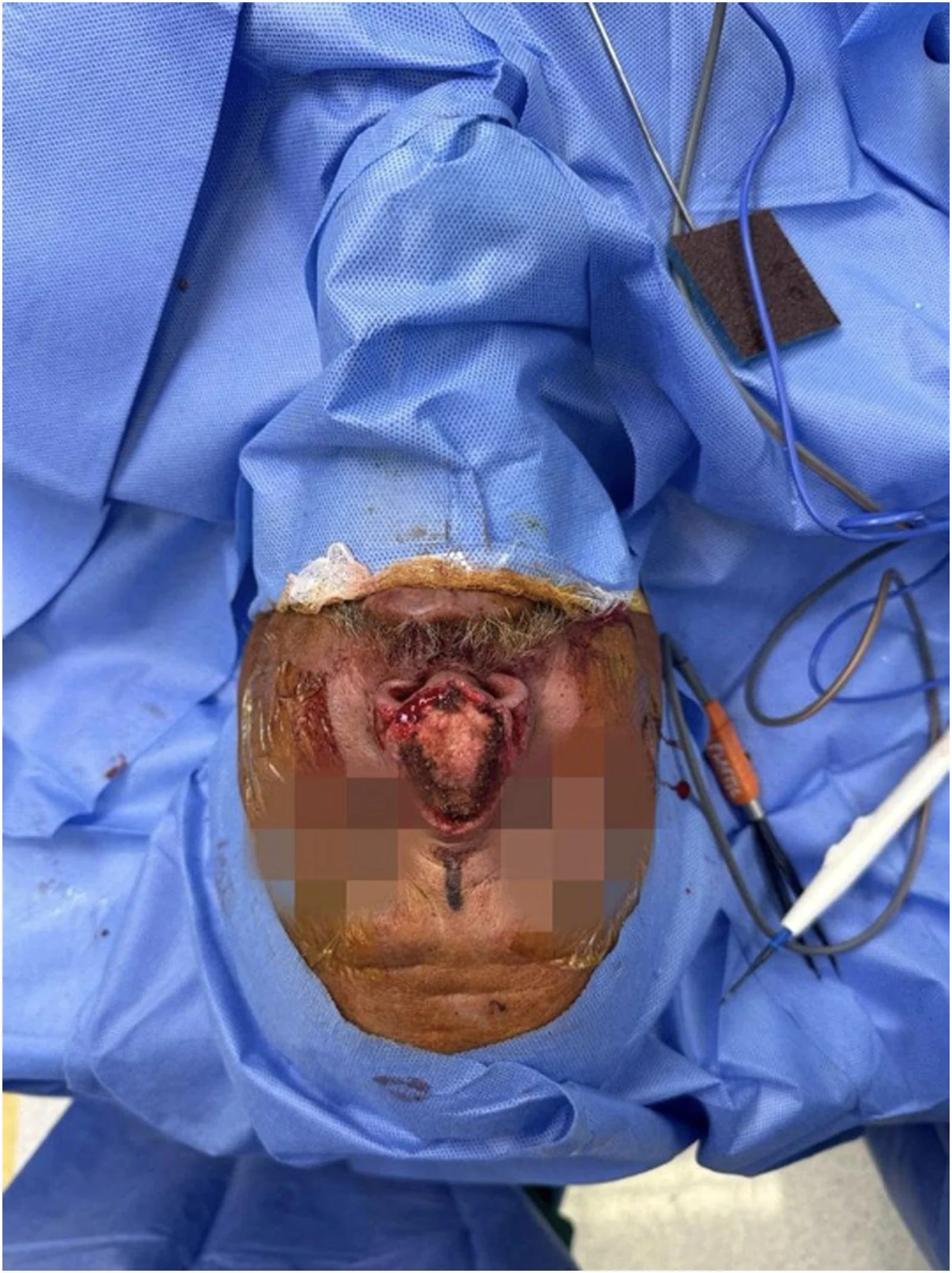
Intraoperative overhead view showing the immediate post-excision appearance with restoration of the natural nasal contour, a clean hemostatic wound surface, and preserved deep dermal adnexal structures to allow re-epithelialization by secondary intention.

Tangential excision was performed with a No 15 blade combined with monopolar electrocautery through a needle-tip electrode, in successive thin, partial-thickness layers parallel to the skin, sparing the deep dermal pilosebaceous structures that source re-epithelialization (Fig. 2, Fig. 3). The contour was sculpted to recreate the nasal tip, supratip break, and alar crests, with periodic inspection for symmetry. Full-dermal penetration was avoided to prevent full-thickness wounds, scarring, hypopigmentation, and alar retraction, and the aesthetic subunits were preserved. Hemostasis was achieved with fine-tip monopolar electrocautery in brief, focused bursts limited to bleeding points, avoiding prolonged contact to minimize lateral thermal spread into the preserved adnexa. After confirming the contour and complete hemostasis, the wound was cleansed with sterile saline and left to heal by secondary intention. A silver-impregnated antimicrobial dressing (Silvercel) was applied to maintain a moist environment and reduce bacterial colonization, secured in layers of Silvercel and gauze under a Tegaderm film shaped to the nose.

Three technical points deserve emphasis, as they were the principal intraoperative difficulties. First, maintaining a uniform plane across surfaces of changing curvature was demanding: the blade tended to skive superficially over the convexity of the tip and to bite too deeply at the alar groove. This was managed by applying counter-traction with the non-dominant hand to flatten the segment being cut, by taking successive thin passes rather than a single deep sweep, and by reorienting the direction of the stroke so that it remained tangential to the local curvature. The correct depth was judged by the appearance of the yellow-white, granular sebaceous lobules studding the wound bed; loss of that appearance, leaving a smooth glistening surface, was taken as the signal to stop in that area. Second, intraoperative assessment of symmetry was limited by infiltration oedema and by the supine position, which foreshortens the dorsum. Symmetry was therefore reassessed at intervals by elevating the head of the table to 30–45° to obtain frontal and basal views, by comparing the operative field against the preoperative photographs displayed in theatre, and by simultaneous bimanual palpation of both alar crests, which detected residual bulk that was not evident visually. Third, punctate bleeding from the transected dermal plexus repeatedly obscured the plane and risked over-resection; alternating short excisional passes with brief, focused cautery of individual bleeding points, rather than broad surface fulguration, kept the field clear while limiting thermal injury to the adnexa. Contouring was completed on the more prominent left side first and the right then matched to it, since correcting an asymmetry by further resection is possible whereas replacing over-resected tissue is not.

## Postoperative phase

**Fig. 4:**
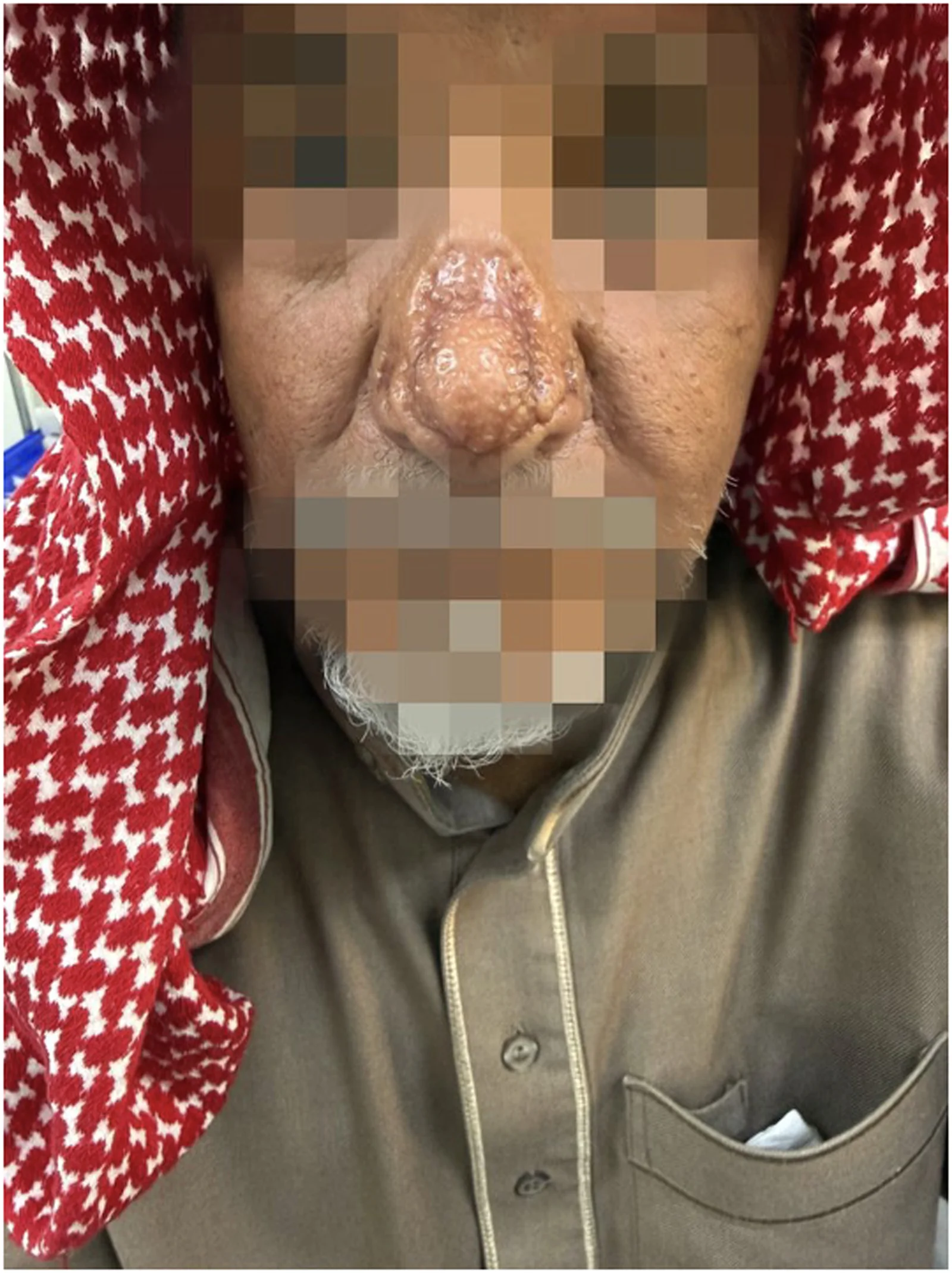
Postoperative frontal view of the final result, showing the well-healed nose with a restored, smooth contour, marked reduction of the rhinophymatous hypertrophy, and a satisfactory aesthetic outcome.

The patient was discharged the same day with instructions on gentle facial hygiene, avoidance of trauma, head elevation during sleep, and follow-up for serial dressing changes. Oral analgesics and prophylactic systemic antibiotics were prescribed to reduce the risk of chondritis and superficial wound infection. At first review he was well, the dressing intact, with no infection or significant bleeding. Subsequent visits showed progressive granulation and gradual re-epithelialization arising from the preserved adnexal islands, consistent with secondary-intention healing. By the 20th postoperative day the site had healed with a smooth contour and no infection (Fig. 4); the silver dressing was replaced by daily topical fusidic acid (Fucidin) over any residual raw areas, and a silicone sheet was applied to optimize scar maturation and reduce erythema and hypertrophic scarring.

Dressing changes followed a defined schedule under clean technique. The first change was performed on postoperative day 2, when the outer gauze and Tegaderm film were removed and the silver-impregnated layer inspected; changes were then carried out every 48 to 72 h until day 10, and every 3 to 4 days from day 10 to day 20 as exudate declined. At each change the wound was irrigated with sterile normal saline, adherent fibrin and crust were gently removed, and a fresh silver-impregnated layer, gauze, and a Tegaderm film shaped to the nose were reapplied; the granulating surface was not otherwise disturbed and no sharp debridement was required. Exudate was moderate during the first week, minimal in the second, and negligible thereafter. At day 20 two small residual raw areas persisted over the supratip and the left alar crest; these were treated with topical fusidic acid 2% ointment once daily beneath a non-adherent dressing until complete re-epithelialization on postoperative day 27, after which silicone sheeting was continued nightly for 12 weeks. No maceration, contact sensitivity, chondritis, or wound infection occurred, and dressing changes required no analgesia beyond the prescribed oral agents.

## Discussion

Rhinophyma, the severe end of the phymatous rosacea spectrum, causes substantial cosmetic disfigurement and psychosocial morbidity even without pain or functional symptoms.^2^^,^^4^ Our patient’s sex and clinical phenotype fit the recognized epidemiology, and at 71 years of age he lies at the upper end of the usual range.^2^^,^^3^ His hypertension, although relevant to perioperative optimization, has no established pathogenetic link to rhinophyma. Although historically linked to alcohol, this association has been refuted, and the condition occurs independently of alcohol consumption.^10^

Histopathological confirmation, as performed here, both confirms the diagnosis and excludes occult malignancy. Basal cell carcinoma is the most frequent neoplasm coexisting with rhinophyma, although squamous cell carcinoma, sebaceous carcinoma, and rare angiosarcoma and lymphoma have been reported.^8^^,^^9^ The prevalence of incidental basal cell carcinoma varies, from about 1.4% in one large series to up to 3% in others.^8^^,^^9^ Comprehensive assessment of all excised tissue is therefore mandatory, since a partial biopsy may give a false-negative result and miss a focally invasive neoplasm.^8^

Because established disease is refractory to medical therapy, surgery remains the mainstay.^5^ A systematic review found tangential excision and CO₂ laser comparable in cosmetic outcome, but tangential excision affords shorter return-to-social-life times, lower cost, and less hypopigmentation and scar contraction than deep CO₂ ablation,^7^ whereas full-thickness excision with grafting adds donor-site morbidity, graft-failure risk, and inferior color match.^6^ Our technique mirrors that of Al Hamzawi and Al Baaj;^3^ preserving the deep pilosebaceous units allowed spontaneous re-epithelialization within about 2 to 4 weeks, matching our patient’s course. Monopolar electrocautery provided rapid, reliable hemostasis and a clear field; judicious application limited to bleeding points minimized deep thermal injury and consequent hypertrophic scarring or pigmentary change.^5^ The postoperative adjuncts (silver-impregnated dressings, topical fusidic acid, and silicone-sheet therapy) are supported by wound-care evidence and similar protocols.^10^ Recurrence is recognized, particularly after superficial excision, as residual tissue at depth may continue to proliferate.^6^ Long-term follow-up, photoprotection, optimization of any underlying rosacea, and patient education about the disease’s chronic, recurrent nature are therefore essential.

Tangential excision with electrocautery is an established rather than a novel modality, and no claim to a new technique is made here; the intended contribution is of a different kind. First, this report documents the operative decision-making rather than the operation alone: the visual and tactile end points used to judge depth, the manoeuvres that keep the plane tangential across surfaces of changing curvature, and the checks used to assess symmetry under conditions of infiltration oedema. These details are largely absent from existing descriptions, yet they determine whether the technique succeeds in less experienced hands. Second, it sets out a staged wound-care protocol for the open nasal wound with each transition tied to the clinical state of the wound rather than to a fixed calendar. Third, the case illustrates that phymatous change may present in the absence of any preceding or concurrent flushing, erythema, papulopustular rosacea, or alcohol history, so that the absence of these features should not delay diagnosis, biopsy, or referral. Taken together, these observations do not alter the choice of procedure; they add reproducible operative and postoperative detail to a technique whose continued relevance rests on its accessibility.

## Limitations

This report has clear limitations. It describes a single patient with moderate disease treated by one surgical team, so the outcome cannot be generalized, and no direct comparison with laser resurfacing, dermabrasion, hydrosurgery, or graft-based reconstruction was possible. The aesthetic result was judged qualitatively by the surgical team and the patient rather than with a validated instrument or blinded independent assessment, and no objective measure of contour, colour match, or scar quality was obtained. Follow-up remains short relative to the natural history of the disease; because residual deep sebaceous tissue may continue to proliferate, recurrence cannot be excluded and surveillance is ongoing. Finally, the technical observations reported here are descriptive and drawn from a single operation, and require confirmation in a prospective series with standardized outcome measures before any comparative advantage over other modalities could be claimed.

## Conclusion

Rhinophyma is a benign but disfiguring condition warranting early surgical management. Tangential excision with a cold blade and monopolar electrocautery, followed by secondary-intention healing with appropriate dressings is simple, low-cost, and reproducible, and in this patient yielded a satisfactory cosmetic outcome without complication. Histopathological examination of all excised tissue remains essential to exclude occult malignancy, particularly basal cell carcinoma.A single case cannot establish superiority over laser-based or graft-based alternatives. This report is offered not as evidence of a new technique but as a detailed and reproducible account of an established one for centres in which instrument-light management is the practical option. Prospective comparative studies using standardized aesthetic outcome measures and longer follow-up are needed to define its place among the available modalities.

## Declarations

### Ethical approval

This case report was conducted in accordance with the principles of the Declaration of Helsinki. Institutional review board approval was not required for the reporting of a single de-identified case.

### Patient consent

Written informed consent was obtained from the patient for publication of this case report and any accompanying images.

### Reporting guideline

This report was prepared in accordance with the STROBE reporting guideline, as recommended for case reports by JPRAS Open.

## Funding

This research did not receive any specific grant from funding agencies in the public, commercial, or not-for-profit sectors.

## Declaration of competing interest

The authors declare that they have no conflicts of interest.
